# Succession Pattern in Soil Micro-Ecology Under Tobacco (*Nicotiana tabacum* L.) Continuous Cropping Circumstances in Yunnan Province of Southwest China

**DOI:** 10.3389/fmicb.2021.785110

**Published:** 2022-02-03

**Authors:** Dan Chen, Yujie Zhou, Mei Wang, Mehr Ahmed Mujtaba Munir, Jiapan Lian, Song Yu, Kuai Dai, Xiaoe Yang

**Affiliations:** ^1^Ministry of Education Key Laboratory of Environment Remediation and Ecological Health, College of Environmental and Resource Sciences, Zhejiang University, Hangzhou, China; ^2^Yuxi Tobacco Company, Ltd. of Yunnan Province, Yuxi, China

**Keywords:** tobacco cultivating, continuous cropping obstacle, soil micro-ecology, succession pattern, metagenomic sequencing

## Abstract

Continuous cropping obstacle (CCO) is a common phenomenon in agricultural production and extremely threatens the sustainable development of agriculture. To clarify the potential keystone factors causing tobacco (*Nicotiana tabacum* L.) CCO, tobacco plants, topsoil, and rhizosphere soil were sampled from the fields with no, slight, and severe tobacco disease in Dali and Yuxi of Yunnan province in China. The physicochemical properties of topsoil and rhizosphere soil, the phenolic acids (PAs) contents in rhizosphere soil, and elemental contents in topsoil, rhizosphere soil, and tobacco plants were analyzed. Microbial diversity in rhizosphere soil was determined by the metagenomic sequencing method. The results showed that soil pH, texture, cation exchange capacity, organic matter, TC, TN, and available K contents showed a significant difference (*p* < 0.05) in soil physicochemical properties. There was a deficiency of B, K, Mg, and Mn contents in soil and/or tobacco plants. The contents of PAs, especially syringic acid in rhizosphere soil, varied significantly among the three sampling groups (*p* < 0.05). Meanwhile, microbial communities and functional genes changed from beneficial to harmful, showing an intimate correlation with soil pH and syringic acid content. It can be concluded that tobacco CCO could be allocated to the imbalance of soil micro-ecology, which possessed a regional feature at the two sampling sites.

## Introduction

Continuous cropping (CC) in the same field has been widespread in China and even all over the world as the result of the limited soil resources, the driving of economic benefits, and the lack of reasonable cropping concept (Zhang et al., 2013; Yuan et al., 2014). Recently, it has been reported that over 20% of the land in China had displayed severe negative consequences of CC, which is called continuous cropping obstacle (CCO) (Bai et al., 2019). CCO refers to the phenomenon that constant cultivation of the same plants or closely related plants in the same field can cause stunted plant growth, a favorable environment for plant pests and diseases, and the reduction in plant yield and quality, even under normal cultivating circumstances (Zhang et al., 2013). CCO is listed as one of the major problems and challenges in agricultural production. It has caused enormous economic losses annually and threatened agricultural sustainable development, provoking an increasing concern about the problems associated with it (Bonner and Galson, 1944; Qu and Wang, 2008).

There are three main causes of CCO, namely, deterioration of soil physicochemical properties, accumulation of plant allelopathic substances (primarily phenolic acids), and altered soil microbial diversity, which can be collectively referred to as imbalance of soil micro-ecological environment (Zhou and Wu, 2012; Yin et al., 2016; Chen et al., 2018; Bai et al., 2019). What is noteworthy is that these three parts react close to each other and ultimately cause huge economic and ecological losses. Phenolic acids (PAs), classified as small molecular organic substances and secondary metabolites, can destroy the plant antioxidant system (Hiradate et al., 2005), restrain primary and secondary root growth, and the basal respiration of plant roots under a specific concentration (Gao et al., 2009). PAs can damage the mitochondria, plastids, nuclear membrane, and endoplasmic reticulum membrane to various extents, including membrane structure and function changes in plants (Chen et al., 2005; Wu and Ma, 2006). Meanwhile, PAs have negative influences on seed germination and seedling’s normal growth. For instance, Ren et al. (2015) verified that the allelochemicals in tobacco rhizosphere soil played an apparate negative role in seed germination, plant growth, and development of tobacco. Different plant species generate special plant-related types of PAs. The release pathways of PAs into the soil include plant evaporation, leaching, root secretion, and the degradation of litter and residues (Weir et al., 2004). As a result, unique PAs can accumulate in the soil after year’s and year’s cropping of the same plant. This phenomenon has been demonstrated in the rhizosphere soil of CC plants, such as tobacco (Bai et al., 2019; Chen et al., 2019). When PAs enter the soil, they can change microbial community structure and diversity as C/N sources. Qu and Wang (2008) found that -phenol 2,4-di-tert-butylphenol and vanillic acid influenced microbial biomass, activity, and community composition in an incubation experiment. In a CC field, the microbial communities were changed constantly under exposure to root exudates with crop-specific microorganisms enriched (Chen et al., 2018). Microorganisms in the soil also affect PAs content, persistence, availability, and allelopathy through degradation (Inderjit, 2005). Soil microbes are vital for maintaining soil quality and ecosystem, including the turnover of organic matter (OM), the degradation of toxic substances, the acceleration of nutrient availability, and the improvement of stress tolerance to pathogens to regulate soil-borne diseases (Brussaard et al., 2007a,b). Henceforth, soil microbial communities are crucial to plant establishment and normal growth (Epelde et al., 2010).

Several studies have shown that long-term monocropping leads to changes in the soil microbial community composition, structure, activity, and function in many plants sensitive to CC (Chen et al., 2018; Gao et al., 2019; Liu et al., 2019; Pang et al., 2021). It revealed that the soil bacterial community structure changed significantly under long-term CC of tobacco (*Nicotiana tabacum* L.). The bacterial diversity also reduced with the duration of cropping (Bai et al., 2019). Pang et al. (2021) found that different Kyoto Encyclopedia of Genes and Genomes (KEGG) pathways were accumulated in different CC years of sugarcane. The bacteria in rhizosphere soil related to nitrogen and sulfur cycling decreased, and the pathogenic bacteria enriched (Pang et al., 2021). Tobacco is typical among these plants sensitive to CC. It has been used as a model crop in many fundamental studies, including agriculture, biology, and medicine (Echeverria and Zeitlin, 2012; Nielsen et al., 2012; Sierro et al., 2014). It is well known that nicotine is unique in the components of tobacco. The occurrence of Parkinson’s and Alzheimer’s diseases can decline under the action of nicotine, which can regulate the central nervous system when entering a human being’s body (Echeverria and Zeitlin, 2012; Nielsen et al., 2012). Recently, about one-third of the world’s tobacco has been planted in China, and the tobacco industry in China has attributed to approximately 10% of total Chinese revenue as the maximal single revenue source (Zou et al., 2018). Meanwhile, tobacco has been the dominating income source for millions of farmers in China as an economic crop, especially in poor areas, such as in Yunnan and Guizhou province. The tobacco industry plays an essential role in poverty alleviation regions (Hu et al., 2010). However, CCO in tobacco cultivation is also commonly associated with stunted growth, decreased yield, poor quality, and the occurrence of a wide range of destructive soil-borne diseases due to CC (Niu et al., 2017; Chen et al., 2018). It has caused huge economic losses and constrained intensive production.

Although the studies concerning microbial change under CC have widely existed, they usually only focus on the bacterial or/and fungal community composition and diversity, without considering other microorganisms and their functions in soil. Therefore, it is preferable and advised to take the entire microorganisms into consideration under the technology of metagenome sequencing. It can provide us with more information, more accurate results, and maybe new discoveries. Meanwhile, limited studies focus on a soil micro-ecology perspective, and it is unclear that the reasons for CCO in tobacco are coherent or not at different places. The objectives of the present study are the following: (1) to explore the factors that have significant differences and their succession pattern with different levels of tobacco disease under CC circumstances; (2) to further find the key factors contributing to different disease groups; and (3) to elucidate the relationship between the crucial factors and the underlying mechanisms causing tobacco CCO from a soil micro-ecology perspective at the last stage.

## Materials and Methods

### Soil and Tobacco Sampling

Yunnan province is the largest province of tobacco yield and cultivating area in China. We collected two soil and tobacco varieties from Yunnan province with a tropical and subtropical plateau climate, paddy soil cultivating Honghuadajinyuan from Dali city, and red soil cultivating K326 from Yuxi city. Three adjacent fields with the constant natural environment, management methods, and different levels of tobacco disease were chosen in each type of soil, including non-diseased (the disease rate < 5%, named as D1 in Dali and Y1 in Yuxi), slightly diseased (the disease rate approximately 20%, named as D2 in Dali and Y2 in Yuxi), and severely diseased (the disease rate > 70%, named as D3 in Dali and Y3 in Yuxi) groups. The basic information about the sampling groups is listed in Supplementary Table S1. The two varieties of tobacco were cultivated according to the local optimal production technology. Twelve tobacco plants of uniformly same size were chosen, and roots were carefully dug up. The non-rhizosphere soil (attached to the root surface loosely) was removed by shaking heavily, and the rhizosphere soil (0–5 mm away from the root) of four tobacco plants was gently collected to form one sample. Immediately, about 50 g of rhizosphere soil per sample was put into an incubator with ice bags, taken back to the laboratory, and put into the -80°C refrigerator for the determination of PAs contents and microbial metagenome. The remaining rhizosphere soil was subjected to analysis of the physicochemical properties and elemental contents. Meanwhile, we collected the four tobacco plants for the measurement of elemental contents. Topsoil (0–20 cm) near the four chosen tobaccos was collected to form a 1-kg mixed topsoil sample. There were three independent repetitions per field for a total of 18 tobacco root, 18 tobacco stem, 18 tobacco leaf, 18 topsoil, and 18 rhizosphere soil samples.

### Determination of Soil Physicochemical Properties

The soil was air-dried, ground, and sieved to pass through a 2-mm mesh for the analysis of soil pH, texture, available K, available P, and nitrate-nitrogen (NO_3_-N) contents, and through a 0.15-mm sieve for the determination of OM, cation exchange capacity (CEC), and total carbon and nitrogen (TC and TN) contents. The soil pH was analyzed by a pH parameter (soil:water = 1:2.5, Multiparameter SevenExcellence, Shanghai, China) (Bao, 2000). The available K was extracted in ammonium acetate solution and evaluated by an atomic absorption spectrometer (AAS, Analytik Jena novAA 300, Germany) method (Bao, 2000). The available P in acid and alkaline soil was extracted in HCl-H_2_SO_4_ and NaHCO_3_ solution, respectively, and then determined by the ultraviolet spectrophotometer (UV-1890, Daojin Instrument Co., Ltd., Jiangsu, China) method (Bao, 2000). The NO_3_-N was extracted in potassium chloride solution and determined by the ultraviolet spectrophotometer (UV-1890, Daojin Instrument Co., Ltd., Jiangsu, China) method (Ministry of Agriculture of the People’s Republic of China GB/T 32737-2016, 2016). The bulk density of soil was analyzed by cutting rings (Ministry of Agriculture of the People’s Republic of China NY/T 1121.4-2006, 2006). Soil texture was measured according to the hydrometer method (Ministry of Agriculture of the People’s Republic of China NY/T 1121.3-2006, 2006). The OM was determined through the potassium dichromate-sulfuric acid method (Bao, 2000). The extraction of soil in hexammine cobalt trichloride solution was performed to analyze CEC by the ultraviolet spectrophotometer (UV-1890, Daojin Instrument Co., Ltd., Jiangsu, China) method (Ministry of Environmental Protection of the People’s Republic of China HJ 889-2017, 2017). The TC and TN contents in topsoil and rhizosphere soil were determined through an elemental analyzer (Elemental Vario EL Cube, Germany).

### Determination of Elemental Contents in Topsoil, Rhizosphere Soil, and Tobacco

Plant and soil samples (0.1–0.2 g) were digested with HNO_3_-H_2_O_2_ (5:1, v/v) and HNO_3_-HClO_4_-HF (5:1:1, v/v/v), respectively (Bao, 2000). The digestion solutions were diluted with ultrapure water, then filtered through 0.45-μm filters. Elemental (K/Ca/Mg/S/Fe/B/Mn/Mo/Zn/Cu) contents in the filtrate were determined by an inductively coupled plasma optical emission spectrometer (ICP-OES, iCAP 6000 series, Thermo Scientific, United States). Certified soil reference sample GBW-07401 (GSS-1) and GBW-07405 (GSS-5, National Standard Detection Research Center, Beijing, China) and plant reference sample GBW-100351 (National Research Center for Certified Reference Materials of China) were included in the digestion procedure.

### Determination of Phenolic Acids Contents in Rhizosphere Soil

The method for the determination of phenolic acids contents referred to Tan et al. (2008) with slight modification. Moist rhizosphere soil (15 g) was set overnight with 15 ml 1M NaOH and then was shaken at 210 rpm at 25°C for 30 min the next day. The suspension was centrifuged at 8,000 × *g* for 10 min. Ten milliliters of supernate was acidified with 12M HCl to pH 2.5 and then was set for 2 h for humic acid precipitation. After that, the suspension was centrifuged at 8,000 × *g* for 10 min, and the supernate was passed through a 0.22-μm organic filter subjected to ultra-performance liquid chromatography (UPLC, Agilent 1290, Agilent Technologies Inc., United States). The UPLC analytical conditions for PAs were as follows: chromatographic column, C_18_ (Shimadzu Inert Sustain, 4.6 × 250 mm); column temperature, 40°C; detector wavelength, 280 nm; flow velocity, 1 ml/min; and injection volume, 10 μl. The mobile phase consisted of 0.1% phosphoric acid solution (A-phase) and acetonitrile (B-phase). Seventeen types of standard PAs samples (gallic acid, phthalic acid, *p*-hydroxybenzoic acid, caffeic acid, vanillic acid, vanillin, benzoic acid, coumalic, salicylic acid, ferulic acid, sinapic, benzothiazole, *trans*-cinnamic acid, diethy phthalate, benzyl benzoate, 4-methylphenyl benzoate, and syringic acid) were measured for the retention time and peak size under a certain concentration. The kinds and concentrations of PAs in rhizosphere soil samples were identified by comparing retention time and peak size with respective standards.

### Microbial Metagenome Analysis in Rhizosphere Soil

#### Extraction and Purification of Soil Microbial DNA

The rhizosphere soil in non-diseased and severely diseased groups from Dali and Yuxi was subjected to soil microbial metagenome analysis. There were a total of 12 samples. Rhizosphere soil (0.5 g) was weighted for DNA extraction. According to the manufacturer’s protocol, the total DNA of soil was extracted by following the instructions of PowerSoil^®^ DNA Isolation Kit (Qiagen Gmbh, Qiagen Strasse 1, 40724 Hilden, Germany). The purity and concentration of DNA were determined based on the 260/280 and 260/230 nm ratios through a micro-spectrophotometer (Nano-300, Allsheng, Hangzhou, China). DNA integrity was determined by 1.0% agarose gel electrophoresis and visualized. The sufficient qualified DNA was used for the library preparation and metagenomic sequencing on the Illumina novaseq6000 platform (NEB, United States).

#### Genome Assembly, Non-redundant Gene Catalog Construction, and Gene Function Annotation

We filtered raw tags to get clean tags by using Trimmomatic v0.33, with a quality cutoff of 20. The reads shorter than 100 bp were discarded from the sample. Simultaneously, the reads that were likely to originate from the host were removed using Bowtie2 v2.2.4, referencing the National Center for Biotechnology Information (NCBI) tobacco genome sequences. The remaining high-quality reads of all samples (6.26–7.19 Gb per sample) were taken together and assembled into contigs using MEGAHIT v1.1.2 (Li et al., 2015). We discarded the contigs shorter than 300 bp and assessed the assembly results through QUAST v2.3 (Gurevich et al., 2013). MetaGeneMark v3.26^1^ was used for genes prediction from the contigs (Zhu et al., 2010). Predicted genes from all samples were gathered together to form a large gene catalog. The non-redundant gene catalog was constructed by using CD-HIT v4.6.6^2^ (Fu et al., 2012). Any two genes with over 95% similarity and 90% coverage of the shorter one were picked out, and subsequently, the shorter one was removed from the large gene catalog. Taxonomic and functional annotations were performed by Diamond v0.9.24 combined with KEGG database (2017-03, blastp, *e*-value ≤ 1e-5^3^) (Kanehisa et al., 2004). The KOs were classified into higher KEGG categories and KEGG pathways.

### Statistical Analysis

Statistical analysis, except for microbial data, was performed by SPSS 26.0. One-way ANOVA analysis of variance followed by Duncan’s test was carried out on the data at the level of significance (*p* < 0.05). For the beta diversity of microbial communities and functional genes, permutational multivariate analysis of variance (PERMANOVA) was used to determine the significance of principal coordinate analysis (PCoA) through the Bray–Curtis dissimilarity. The significance of Bray–Curtis non-metric multidimensional scaling (nMDS) was measured by analysis of similarities (ANOSIM) test. The R package of metagenomeSeq was used to assess the relative abundance of the microbial community at the phyla level and functional genes based on the KEGG metabolic pathways (Segata et al., 2011). The biomarkers among different groups were gained from linear discriminant analysis effect size (LEfSe) and random forest analyses. The correlation between the relative abundance of microbial communities, functional genes, and soil environmental factors was analyzed by correlation heatmap and redundancy analysis (RDA). PCoA, nMDS, metagenomeSeq, LEfSe, random forest, correlation heatmap, and RDA were performed by R 3.1.1. PERMANOVA and ANOSIM tests were achieved by QIIME 1.8.0 (Caporaso et al., 2010).

## Results

### Soil Physicochemical Properties

The results of soil physicochemical properties are presented in Table 1. The pH in the topsoil and rhizosphere soil from Dali increased significantly (*p* < 0.01) with the increase in disease rate. However, the topsoil and rhizosphere soil from Yuxi showed an opposite tendency. The OM content increased with the increase in disease rate, with a more evident phenomenon in the soil from Yuxi, which showed an extremely remarkable difference (*p* < 0.01) among the three groups. For the NO_3_-N content, there was no apparent pattern in the topsoil or rhizosphere soil from Dali or Yuxi. The available P content in the topsoil and rhizosphere soil showed a considerable increase (*p* < 0.05) in the severely diseased group from Dali, with no such phenomenon in the soil from Yuxi. The available K content in the topsoil decreased apparently in the severely diseased groups. However, it showed an opposite trend in the rhizosphere soil from Dali and Yuxi. The CEC was higher in the diseased groups than in the non-diseased groups from Dali and Yuxi. There was a negative correlation between bulk density and tobacco disease rate in the topsoil from Yuxi but not in the topsoil from Dali. Simultaneously, the topsoil had more sand and less silt component in the diseased group from Yuxi but owned less sand and more clay in the diseased group from Dali. In general, the contents of TC and TN in the topsoil and rhizosphere soil were higher in the severely diseased groups than in the non-diseased groups from Dali and Yuxi, and the rhizosphere soil showed a more pronounced trend than the topsoil.

**TABLE 1:** The physicochemical properties in the topsoil and rhizosphere soil from Dali and Yuxi.

	pH	OM (g/kg)	NO_3_-N (mg/kg)	Available P (mg/kg)	Available K (mg/kg)	CEC (cmol^+^/kg)	TC (%)	TN (%)	Bulk density (g/cm^3^)	Sand (%)	Silt (%)	Clay (%)
D1	6.07^b^	34.55^a^	56.33^ab^	47.10^b^	261.89^a^	10.34^c^	2.76^b^	0.27^b^	1.27^a^	6.65^a^	55.92^a^	37.43^c^
D2	7.25^a^	35.63^a^	77.91^a^	20.28^c^	165.38^b^	17.08^a^	2.68^b^	0.29^a^	1.29^a^	3.53^c^	54.13^b^	42.34^a^
D3	7.32^a^	36.28^a^	17.72^b^	61.51^a^	211.17^ab^	14.63^b^	2.90^a^	0.28^ab^	1.30^a^	5.27^b^	55.78^a^	38.96^b^
Y1	7.65^a^	26.96^c^	8.77^ab^	54.23^a^	165.51^b^	13.21^c^	2.97^a^	0.18^c^	1.18^a^	5.70^c^	59.41^a^	34.90^a^
Y2	7.33^a^	40.22^b^	4.23^b^	58.06^a^	226.41^a^	28.13^a^	2.97^a^	0.22^b^	1.13^ab^	10.54^b^	50.29^b^	39.16^a^
Y3	6.19^b^	52.04^a^	16.27^a^	55.53^a^	98.18^c^	17.36^b^	3.17^a^	0.26^a^	0.98^b^	19.17^a^	46.45^c^	34.38^a^
DR1	5.93^b^	36.23^b^	4.74^a^	46.67^c^	182.84^b^	9.99^c^	2.77^ab^	0.25^c^	/			
DR2	6.94^a^	35.92^b^	6.67^a^	87.46^b^	281.81^a^	10.14^b^	2.75^b^	0.29^b^				
DR3	6.87^a^	40.80^a^	14.11^a^	111.30^a^	277.05^a^	15.02^a^	3.05^a^	0.29^a^				
YR1	7.79^a^	41.51^b^	9.99^a^	48.37^a^	106.39^b^	13.59^c^	3.06^b^	0.18^c^	/			
YR2	7.30^a^	46.77^b^	6.41^a^	42.40^a^	200.96^ab^	22.98^a^	2.92^b^	0.22^b^				
YR3	6.38^b^	59.44^a^	12.74^a^	51.36^a^	249.38^a^	18.10^b^	3.38^a^	0.26^a^				

*D1, D2, and D3 represent the topsoil in non-, slightly, and severely diseased groups from Dali, respectively; those followed by “R” represent the corresponding rhizosphere soil. Y1, Y2, and Y3 represent the corresponding samples from Yuxi. Different letters show a significant difference within a column in the physicochemical properties of topsoil or rhizosphere soil based on the Duncan’s method (p < 0.05).*

### Elemental Contents in Topsoil, Rhizosphere Soil, and Tobacco

**FIGURE 1:**
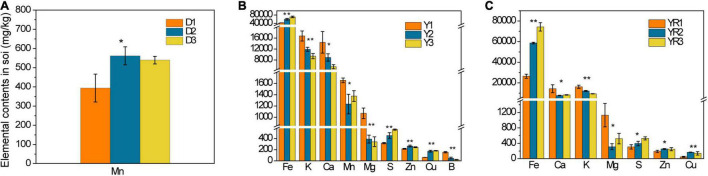
The elemental contents in the topsoil from Dali **(A)** and Yuxi **(B)** and in the rhizosphere soil from Yuxi **(C)**. Values are the average of three replicates with a standard deviation bar. D1, D2, and D3 represent the topsoil in non-, slightly, and severely diseased groups from Dali, respectively. Y1, Y2, and Y3 represent the corresponding samples from Yuxi; those followed by “R” represent the corresponding rhizosphere soil. “**” and “*” represent the contents of the elements showing extremely significant (*p* < 0.01) and significant (*p* < 0.05) difference, respectively.

**FIGURE 2:**
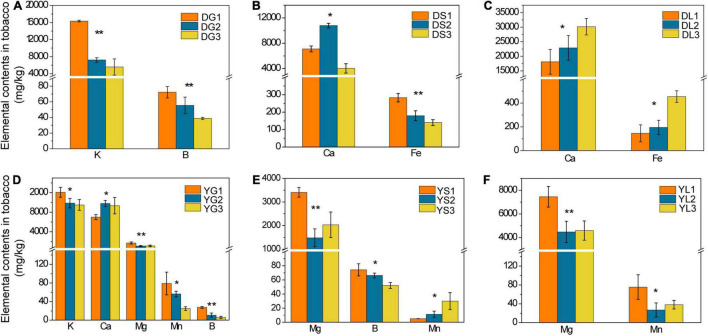
The elemental contents in tobacco roots, stems, and leaves from Dali (**A–C**, respectively) and Yuxi (**D–F**, respectively). Values are the average of three replicates with a standard deviation bar. DG, DS, and DL represent tobacco roots, stems, and leaves from Dali, respectively; those followed by “1,” “2,” and “3” represent tobacco showing no, slight, and severe disease, respectively. YG, YS, and YL represent the corresponding samples from Yuxi. “**” and “*” represent the contents of the elements showing extremely significant (*p* < 0.01) and significant (*p* < 0.05) difference, respectively.

We detected 10 kinds of elements (K/Ca/Mg/S/Fe/B/Mn/Mo/Zn/Cu) in topsoil, rhizosphere soil, and tobacco. Only the elements whose contents showed a significant difference (*p* < 0.05) among the three groups are presented (Figures 1, 2). For the soil from Dali, only the content of Mn increased evidently (*p* < 0.05) in the topsoil of the diseased group (Figure 1A). For the soil from Yuxi, there was a declined trend in the contents of B, Ca, K, Mg, and Mn in the topsoil of the diseased group (Figure 1B). The contents of these elements showed a similar tendency in the rhizosphere soil except for B and Mn (Figure 1C). There was an appreciable increase (*p* < 0.05) in the contents of Cu, Fe, S, and Zn in the diseased group’s topsoil and rhizosphere soil (Figures 1B,C).

For the tobacco collected from Dali, there was an apparent declined trend in B and K contents of tobacco roots in the diseased group (*p* < 0.05) (Figure 2A). The Ca and Fe contents of tobacco stems decreased, whereas tobacco leaves increased obviously (*p* < 0.05) in the severely diseased group (Figures 2B,C). As for the tobacco collected from Yuxi, the B, K, Mn, and Mg contents of tobacco roots declined evidently in the diseased group (*p* < 0.05) (Figure 2D). The B and Mg contents of tobacco stems decreased obviously in the diseased group, but the Mn content increased apparently (*p* < 0.05) (Figure 2E). There was an obvious decrease in the Mn and Mg contents in tobacco leaves of the diseased group (Figure 2F). The B and K contents in tobacco roots decreased remarkably in the diseased groups both from Dali and Yuxi. There was a marked decrease (*p* < 0.05) in Mg content of tobacco roots, stems, and leaves in the diseased group from Yuxi. Overall, it showed a similar declined trend for B, K, Mn, and Mg contents in the topsoil and roots of the diseased group from Yuxi, without such pattern for Cu, Fe, S, and Zn contents. The differences in the contents of the analyzed elements were more apparent in the soil and tobacco collected from Yuxi than from Dali.

### Phenolic Acids Contents in Rhizosphere Soil

**FIGURE 3:**
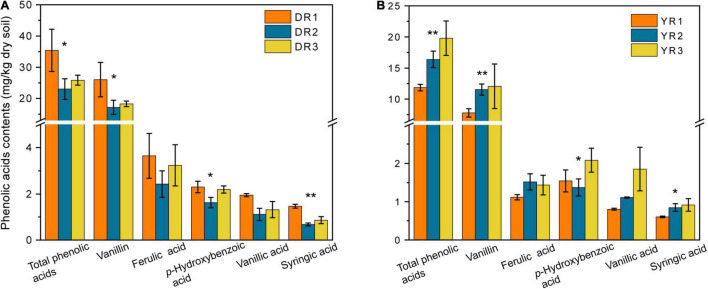
The phenolic acid contents in the rhizosphere soil from Dali **(A)** and Yuxi **(B)**. Values are the average of three replicates with a standard deviation bar. DR1, DR2, and DR3 represent the rhizosphere soil in non-, slightly, and severely diseased groups from Dali, respectively. YR1, YR2, and YR3 represent the corresponding samples from Yuxi. “**” and “*” represent the contents of the phenolic acids showing extremely significant (*p* < 0.01) and significant (*p* < 0.05) difference, respectively.

Although 17 varieties of standard reference samples of PAs were prepared and determined, only five varieties (syringic acid, vanillic acid, *p*-hydroxybenzoic acid, ferulic acid, and vanillin) could be detected in the rhizosphere soil in our research (Figure 3). The vanillin content in the rhizosphere soil was the highest (7.78–26.03 mg/kg dry soil), followed by the ferulic acid (1.12–3.64 mg/kg dry soil), *p*-hydroxybenzoic acid (1.37–2.30 mg/kg dry soil), and vanillic acid (0.81–1.95 mg/kg dry soil). The syringic acid content was the lowest (0.60–1.47 mg/kg dry soil). The syringic acid and vanillic contents in the rhizosphere soil showed a considerable decrease (*p* < 0.05) in the diseased group from Dali. However, they showed an opposite pattern from Yuxi. Overall, there was a significant decreasing trend in the total content of the five PAs in the diseased group from Dali (*p* < 0.05), whereas the rhizosphere soil from Yuxi showed an opposite pattern.

### Analysis of Microbial Community in Rhizosphere Soil

After the metagenomic sequencing, we obtained over 15,430,074 clean reads per sample (Supplementary Table S2). While in metagenomic assembling, we gained 84,962–193,544 contigs with the mapped ratio ranging from 37.11 to 48.97% per sample after excluding the contigs < 300 bp (Supplementary Table S3). Using MetaFeneMark software for gene prediction, over 445,679 genes were obtained per sample (Supplementary Table S4). There were a total of 6,596,898 genes with an average length of 360 bp in the non-redundant gene set based on the CD-HIT software (Supplementary Table S5).

#### Composition and Relative Abundance of Microbial Community

**FIGURE 4:**
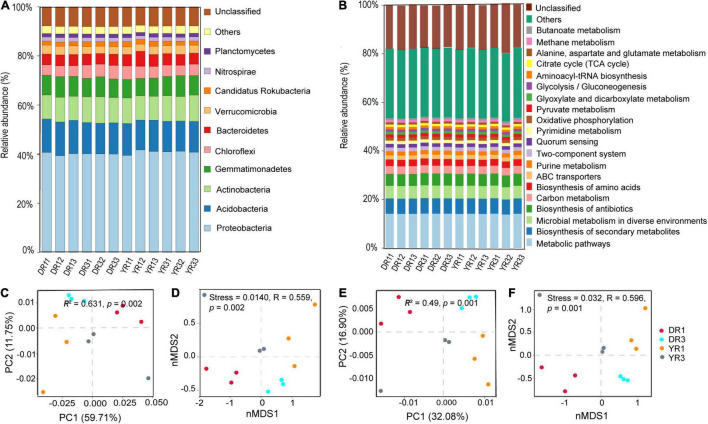
The relative abundance and β-diversity of microbial communities and KEGG pathways. **(A)** The relative abundance of the microbial community at the phylum level. Only the first 10 most dominant microorganisms are presented. **(B)** The relative abundance of KEGG pathways at level 3. Only the first 20 most dominant KEGG pathways are presented. **(C)** Bray–Curtis PCoA and PERMANOVA analysis based on microbial species. **(D)** Bray–Curtis nMDS and ANOSIM analysis based on microbial species. **(E)** Bray–Curtis PCoA and PERMANOVA analysis based on KEGG pathways. **(F)** Bray–Curtis nMDS and ANOSIM analysis based on KEGG pathways. DR1_ and DR3_ (_ = 1, 2, 3) represent the rhizosphere soil in non- and severely diseased groups from Dali, respectively. YR1_ and YR3_ represent the corresponding samples from Yuxi.

There were five kinds of microorganisms at the kingdom level in all rhizosphere soil samples, eukaryote, fungi, viruses, archaea, and bacteria. The bacteria owned the highest relative abundance (81.58–83.78% in all samples), followed by the archaea (0.30–0.39%), viruses (0.18–0.21%), and fungi (0.15–0.47%). The first six highest relative abundance of microorganisms at the phylum level were Proteobacteria (32.88–35.30% in all samples), Acidobacteria (10.18–11.49%), Actinobacteria (8.17–9.04%), Gemmatimonadetes (5.98–7.02%), Chloroflexi (3.48–4.63%), and Bacteroidetes (3.63–5.17%), with the average total relative abundance of 67.69% (Figure 4A). The first five highest relative abundance of microorganisms at the species level were *Acidobacteria bacterium* (5.22–6.18% in all samples), *Gemmatimonadetes bacterium* (2.95–3.71%), *Chloroflexi bacterium* (1.51–2.17%), *Verrucomicrobia bacterium* (1.31–1.73%), and *Candidatus Rokubacteria bacterium* (0.91–1.36%), with the average total relative abundance of 13.45% (Supplementary Figure S1).

#### Beta Diversity and Difference Analysis of Microbial Community

The Bray–Curtis PCoA and PERMANOVA analysis based on species showed that the severely diseased group was remarkably distinct from the non-diseased group, with the first two axes explaining 59.71 and 11.75% variance (*R*^2^ = 0.631, *p* = 0.002) (Figure 4C). This phenomenon could also be verified from Bray–Curtis nMDS and ANOSIM analysis based on species (Figure 4D), which showed remarkable variations between the two groups from Dali and Yuxi (stress = 0.014, *R* = 0.559, *p* = 0.002). The distance between the two groups from Dali was farther than from Yuxi, presenting a more pronounced difference in β-diversity of microbiome from Dali than from Yuxi.

**FIGURE 5:**
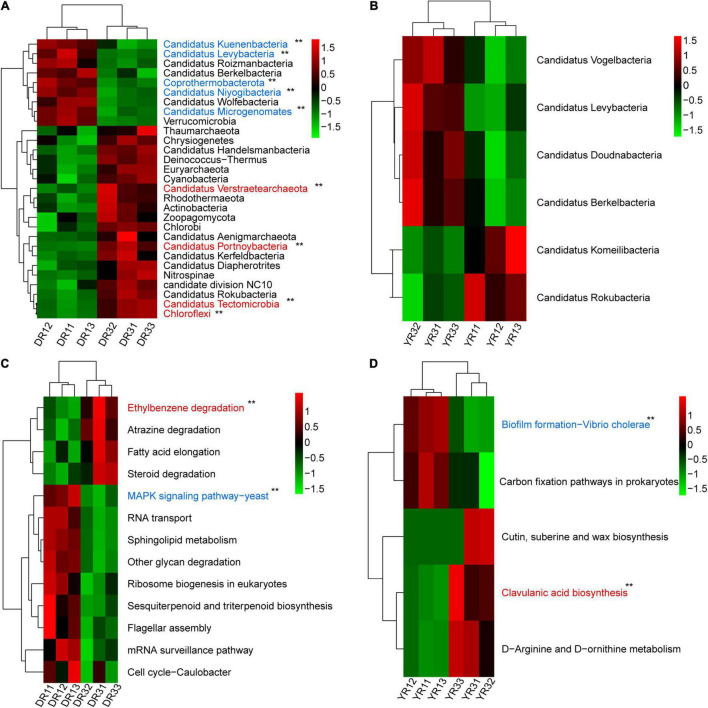
The heatmap of difference analysis of microbial communities and KEGG pathways. **(A,B)** The heatmap of the relative abundance of microorganisms at the phylum level in Dali **(A)** and Yuxi **(B)**. **(C,D)** The heatmap of the relative abundance of KEGG pathways in Dali **(C)** and Yuxi **(D)**. Only the microorganisms and KEGG metabolic pathways are presented whose relative abundance showed a significant difference (*p* < 0.05). The *p*-value of the microbiome and KEGG pathways marked with “**” was <10^–4^ based on the significance analysis, with the blue one enriched in the non-diseased group and the red one enriched in the severely diseased group. DR1_ and DR3_ (_ = 1, 2, 3) represent the rhizosphere soil in non- and severely diseased groups from Dali, respectively. YR1_ and YR3_ represent the corresponding samples from Yuxi.

**FIGURE 6:**
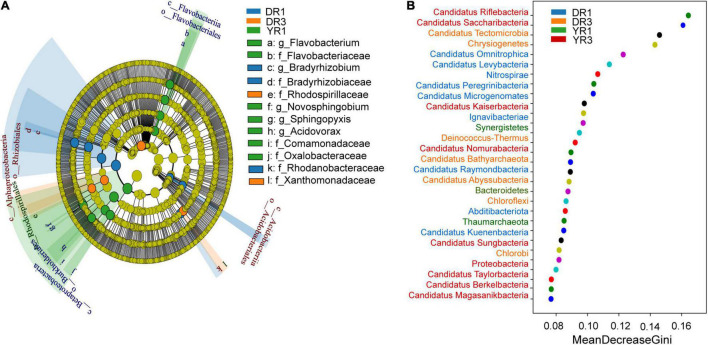
The LEfSe and random forest analyses of microbial communities. **(A)** The cladogram of the microorganisms based on the LEfSe analysis. The threshold value of LDA is 2. The blue, orange, and green taxonomies represent the biomarkers playing an important role in the non- and severely diseased group from Dali and non-diseased group from Yuxi, respectively. No such taxonomy existed in the severely diseased group from Yuxi. The relative abundance of the yellow one showed no significant difference (*p* ≥ 0.05). **(B)** The random forest analysis of the microorganisms at the phylum level. DR1 and DR3 represent the rhizosphere soil in the non- and severely diseased group from Dali, respectively. YR1 and YR3 represent the corresponding samples from Yuxi.

From the metagenomeSeq analysis, the heatmap of the relative abundance of the microbial community at the phylum level was divided into two parts evidently between the two groups from Dali and Yuxi (Figures 5A,B). Candidatus Kuenenbacteria, Candidatus Levybacteria, Coprothermobacterota, Candidatus Niyogibacteria, and Candidatus Microgenomates were enriched in the non-diseased group in Dali (Figure 5A). Candidatus Verstraetearchaeota, Candidatus Portnoybacteria, Candidatus Tectomicrobia, and Chloroflexi were enriched in the diseased group from Dali (*p* < 10^–4^) (Figure 5A). There was no such phylum existing in the rhizosphere soil from Yuxi (*p* > 10^–4^) (Figure 5B). In every group, except for the diseased group from Yuxi, there existed some biomarkers contributing to group difference based on the LEfSe analysis (Figure 6A). It showed that the first two most important biomarkers per group were class Alphaproteobacteria and order Phizobiales in the non-diseased group, and were phylum Chloroflexi and family Xanthomonadaceae in the diseased group from Dali (Supplementary Figure S2). Meanwhile, they were class Betaproteobacteria and order Burkholderiales in the non-diseased group from Yuxi (Supplementary Figure S2). From the random forest analysis, Candidatus Omnitrophica and Candidatus Levybacteria (enriched in the non-diseased group) and Candidatus Tectomicrobia and Chrysiogenetes (enriched in the diseased group) were the most apparent biomarkers from Dali (Figure 6B). They were Synergistetes and Bacteroidetes (enriched in the non-diseased group) and Candidatus Riflebacteria and Candidatus Saccharibacteria (enriched in the diseased group) from Yuxi (Figure 6B).

#### Correlation Analysis Between Microbial Community and Environmental Factors in Rhizosphere Soil

**FIGURE 7:**
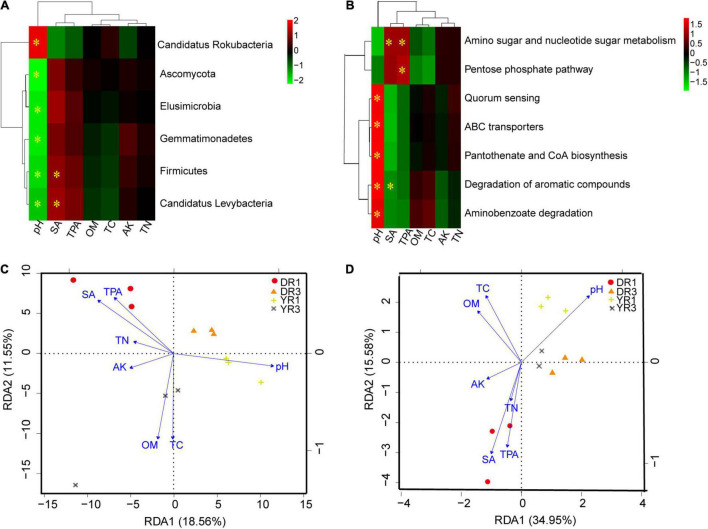
The correlation analysis between microorganisms or KEGG pathways and soil environmental factors. **(A**,**B)** The correlation heatmap between microorganisms at the phylum level **(A)** or KEGG pathways **(B)** and soil environmental factors. The box marked with “*” showed a significant difference (*p* < 0.05). **(C**,**D)** The RDA analysis between microorganisms at the species level **(C)** or KEGG pathways **(D)** and soil environmental factors. SA, TPA, OM, TC, AK, and TN represent the contents of syringic acid, total phenolic acids, organic matter, total C, available K, and total N in rhizosphere soil. DR1 and DR3 represent the rhizosphere soil in the non- and severely diseased groups from Dali, respectively. YR1 and YR3 represent the corresponding samples from Yuxi.

Based on the correlation heatmap between soil microbial community at the phylum level and environmental factors in rhizosphere soil, soil pH and syringic acid (SA) were the most important factors, showing an obvious correlation (*p* < 0.05) with more microorganisms (Figure 7A and Supplementary Figure S3A). Noticeably, the relative abundance of Firmicutes and Candidatus Levybacteria had a pronounced positive relationship (*p* < 0.05) with the content of SA. One microbial group showed a positive relationship with soil pH. Simultaneously, it showed a negative interaction with SA or total phenolic acids (TPAs) (Supplementary Figure S3A). Based on the RDA analysis between microbial community at the species level and soil environmental factors, the first two components explained 18.56 and 11.55% of the total variance (Figure 7C). The three samples in the same group were grouped on a whole view, and different groups were separated primarily on the first component (RDA1). Soil pH, OM, TC, SA, and TPA contents were the most crucial environmental factors influencing the microbial community. For the non-diseased group from Dali, SA, TPA, TN, and AK had a positive effect, whereas soil pH, OM, and TC had a negative influence. Interestingly, there was an opposite phenomenon in the non-diseased group from Yuxi. Meanwhile, OM and TC showed a positive relationship with the diseased group from Yuxi.

### Analysis of Microbial Functional Genes in Rhizosphere Soil

#### Composition and Relative Abundance of Functional Genes

Based on the KEGG analysis of functional genes, the relative abundance of KEGG pathways at level 3 in the samples is listed in Figure 4B. The first four dominant KEGG pathways are metabolic pathways (13.8–14.20% in all samples), biosynthesis of secondary metabolites (6.19–6.37%), microbial metabolism in diverse environments (5.05–5.28%), and biosynthesis of antibiotics (4.87–5.01%) (Figure 4B).

#### Beta Diversity and Difference Analysis of Functional Genes

The Bray–Curtis PCoA and PERMANOVA analysis at the KO level showed a distinct functional gene pattern in the non-diseased groups from the diseased groups, with the first two axes depicting 32.08 and 16.90% of the total shift (*R*^2^ = 0.49, *p* = 0.001) (Figure 4E). The Bray–Curtis nMDS and ANOSIM analysis further confirmed this phenomenon (stress = 0.032, *R* = 0.596, *p* = 0.001) (Figure 4F). The difference in the β-diversity of the functional genes was more remarkable between the two groups from Dali than from Yuxi. The metagenomeSeq analysis based on the KEGG metabolic pathways showed that the heatmap of the relative abundance of functional genes was divided into two parts between the non-diseased and the diseased groups in Dali and Yuxi (Figures 5C,D). The abundance of genes related to mitogen-activated protein kinase (MAPK) signaling pathway yeast and ethylbenzene degradation showed a significantly higher abundance in the non-diseased and diseased groups in Dali, respectively (*p* < 10^–4^). In comparison the genes concerning biofilm formation in *Vibrio cholerae* and clavulanic acid biosynthesis were enriched in the non-diseased and diseased groups in Yuxi, respectively (*p* < 10^–4^).

#### Correlation Analysis Between Microbial Functional Genes and Environmental Factors in Rhizosphere Soil

From the correlation heatmap between the relative abundance of functional genes and soil environmental factors, soil pH, SA, and TPA owned a more marked correlation with functional genes than OM, TC, AK, and TN (Figure 7B and Supplementary Figure S3B). The correlation pattern between functional genes and SA was similar to that between functional genes and TPA, which was opposite between functional genes and soil pH (Supplementary Figure S3B). Based on the RDA analysis, the first (34.95%) and the second axes (15.58%) explained 50.53% of the total variance (Figure 7D). Soil pH, SA, and TPA were the most important environmental factors contributing to the functional gene difference. The three samples from the same group were gathered, and the two diseased groups in Dali and Yuxi were assembled. However, the two non-diseased groups were separated.

## Discussion

The deterioration of soil physicochemical properties is a vital cause of CCO (Zhang et al., 2016; Chen et al., 2018; Bai et al., 2019). Plants absorb nutrients directly from the soil to maintain normal life activities. Simultaneously, soil provides microorganisms with nutrient, energy sources, and a suitable living environment. The microbial diversity changes with the variance in soil physicochemical properties (Chen et al., 2018; Bai et al., 2019). Based on the significance analysis, soil pH, OM, available K, CEC, TC/TN contents, and texture were the main parameters showing differences between the two groups from Dali and Yuxi. Soil acidification is a common phenomenon in long-term CC soil (Zhang et al., 2016; Shen et al., 2018; Bai et al., 2019). Soil pH is closely correlated with tobacco soil-borne diseases, with a lower value in the diseased than non-diseased fields (Li et al., 2017). It is presented in the soil from Yuxi in the present study. However, a reverse result is observed in the soil from Dali, with a higher pH in the diseased group. It has been proposed that cation exchange and mineral weathering are two main ways for proton (H^+^) consumption in soil acid-buffering mechanisms (Yang et al., 2013). The two H^+^-consumption processes may have played a more dominant role than the H^+^-releasing processes in the soil from Dali. The different microbial communities in the soil also contributed to the different change pattern of soil pH at the two sites. Soil organic matter (SOM) plays an important role in maintaining soil ecology as water conservation and providing plants and microbes with nutrients (Obalum et al., 2017). Interestingly, the OM content showed an increasing trend in the diseased groups, which was opposite to the results of other studies. Chen et al. (2018) found that the SOM content and soil fertility decreased in the tobacco CC field. Bai et al. (2019) discovered that soil pH, OM, and available P contents decreased in the CC field of tobacco, whereas the available K content showed an increasing trend over the cropping time. In our study, the available K content in the rhizosphere soil increased with the increase in CC years, which may be ascribed to the accumulation of K fertilizer applied annually, and the destroyed ability of diseased tobacco to absorb K^+^ from the rhizosphere soil. We observed a higher CEC in the diseased groups both from Dali and Yuxi, which is coherent with the results of Mareschal et al. (2013). As for the TC and TN contents in the soil, the diseased groups showed an increasing trend in Dali and Yuxi. On the one hand, the nitrogen fertilizers application and the diseased tobacco discarded in the field contributed to the source of C and N. On the other hand, this could be owed to the lower rate of C and N element circle in the diseased groups under the influence of the unbalanced micro-ecology system (Pang et al., 2021). The abundance of genes specific to denitrification like nirK/S, norBC, and nosZ decreased after 30 years of sugarcane continuous cropping (Pang et al., 2021). Although the OM, available K, CEC, and TC/TN contents showed a similar variation trend in the soil from Dali and Yuxi, some parameters still presented an opposite tendency at the two sampling sites, like soil pH, texture, and available P content. This can be owing to the differences in soil types, tobacco varieties, natural conditions, and anthropogenic activities (Bai et al., 2019).

From the analysis of elemental contents in topsoil, rhizosphere soil, and tobacco plants, there was a phenomenon of elemental imbalance in the diseased groups. The Ca and Fe contents declined in tobacco stems but increased in tobacco leaves in the diseased group from Dali. There was a great possibility that Ca and Fe in tobacco stems were transferred to the leaves. The B, K, Mn, and Mg contents decreased consistently in the topsoil and tobacco roots of the diseased group from Yuxi. It was noticed that the deficiency of these elements in tobacco roots could partially originated from the lack of these elements in the soil. Tobacco is favorable in absorbing certain varieties of elements from the soil, and the elemental imbalance will form gradually under long-term CC, improper fertilizer application, and changed processes of soil mineral weathering (Zhang et al., 2016). Elemental imbalance in the soil or/and plants is a common phenomenon under CC circumstances and plays an important role in causing CC problems. For instance, Chen et al. (2018) presented that the content of Fe decreased evidently in the CC tobacco soil. In contrast, B showed an opposite trend, and no significant variance was observed for Ca, Mg, Na, Mn, and Zn. Zhang et al. (2016) found an obvious desilication, weathering of potassium-bearing minerals, and accumulation of Al and Fe under long-term tobacco plantation than under the fallow. Meanwhile, alterations of the mineralogical properties in topsoil is related to change in soil pH (Zhang et al., 2016). In conclusion, B, K, Mg, and Mn played an important role in tobacco disease with a noticeable declining contents in the diseased groups in our study (*p* < 0.05). B is an essential microelement for plants, having an important adjusting function in the formation of cytoderm, cell division, and C and N metabolism (Xiao, 1997; Liu, 2017). B can improve the ability of stress resistance in tobacco by enhancing tobacco root growth. Meanwhile, B influences the yield and quality of tobacco in combination with Ca and K (Xiao, 1997; Liu, 2017). K is a major element that tobacco absorbs most from the soil, and a high content of K in tobacco leaves can promote the quality of tobacco by playing a positive role in the synthesis and accumulation of some aromatic substances (Xiao, 1997; Liu, 2017). It has been verified that K can promote the activation of at least 60 enzymes, therefore can speed up the synthesis of sugar, protein, and photosynthesis by motivating the usage of light energy (Xiao, 1997; Liu, 2017). Potassium fertilizer can improve the stress resistance of tobacco, including drought, salt, disease, and lodging resistance (Xiao, 1997; Liu, 2017). Mg is the component of chlorophylls and is important for the photosynthesis process, which is correlated with many physiological activities as the activator of related enzymes (Xiao, 1997; Liu, 2017). There is a significant possibility that the yield and quality of tobacco decrease for a lack of Mg. Mn is the essential element for plants to form chlorophylls and maintain chlorophylls’ normal structure (Xiao, 1997; Liu, 2017). Simultaneously, Mn is the activator of varieties of enzymes, which take part in photosynthesis and respiration (Xiao, 1997; Liu, 2017). Therefore, the scarcity of B, K, Mg, and Mn has an indispensable relationship with the eruption of tobacco disease in recent research. For this reason, appropriate application of B, K, Mg, and Mn fertilizers is suggested in alleviating tobacco CCO.

In our study, there was an increasing trend in the total contents of ferulic acid, vanillin, syringic acid, vanillic acid, and *p*-hydroxybenzoic acid in the rhizosphere soil of the diseased group from Yuxi, indicating that the accumulation of PAs was a vital factor resulting in tobacco disease from Yuxi. This phenomenon was coherent to the results of most recent studies (Qu and Wang, 2008; Bai et al., 2019). Chen et al. (2019) determined 18 PAs in the rhizosphere soil from the CC tobacco field. They found that *p*-hydroxybenzoic acid, *p*-coumaric acid, vanillic acid, ferulic acid, and syringic acid were the predominant PAs, with the total content in the 30-year CC soil higher than that in the 20-year CC soil. Bai et al. (2019) revealed that the contents of phloroglucinol, coumalic acid, *p*-hydroxybenzoic acid, vanillic acid, and ferulic acid increased with increasing cropping duration of tobacco. In these cases, plant root residues and exudates might provide pathogens with a suitable physical and nutritional environment, thus contributing to the biomass of pathogen fungi. It destroys the balance of bacterial and fungal abundance, with the accumulation of pathogens and the reduction in beneficial microbes (Qu and Wang, 2008; Zhou and Wu, 2012). It should be mentioned that different plants release diverse PAs into the soil. Phlorizin, benzoic acid, and vanillic aldehyde were the predominant phenolic acids in a replanted apple orchard, which maybe causing apple replant diseases (Yin et al., 2016). It was identified that 4-hydroxybenzoic acid, vanillic acid, ferulic acid, benzoic acid, 3-phenylpropionic acid, and cinnamic acid existed in CC cucumber soil (Chen et al., 2011). The impact of PAs on plant growth can be concluded in two aspects: one is to inhibit plants directly by preventing nutrient uptake and causing cell membrane peroxidation *via* allelopathy (Chen et al., 2005; Hiradate et al., 2005; Wu and Ma, 2006), and the other is to suppress plant growth indirectly by changing the soil micro-ecological environment (Wang et al., 2016). However, there was an opposite pattern in the rhizosphere soil from Dali, contrary to the common conclusions. The volatilization and degradation processes in the rhizosphere soil might exert a more dominant effect than that in the plants producing and releasing PAs in Dali. There was a reverse situation in Yuxi.

Our results showed that the microbial composition and structure varied between the non-diseased and diseased groups. The first six dominant phyla in our study were Proteobacteria (32.88–35.30% of all samples), Acidobacteria (10.18–11.49%), Actinobacteria (8.17–9.04%), Gemmatimonadetes (5.98–7.02%), Bacteroidetes (3.63–5.17%), and Chloroflexi (3.48%–4.63%), which was consistent with the results of previous studies (Niu et al., 2016; Bai et al., 2019). We found that the phylum Chloroflexi was enriched in the diseased group from Dali, which was similar to the findings of Chen et al. (2018). Niu et al. (2016) also revealed that Chloroflexi accumulated in the CC tobacco field, and its abundance, was positively related to tobacco disease rate. There is a great possibility that Chloroflexi competes in nitrogen source with tobacco, as Chloroflexi cannot fix nitrogen (Kragelund et al., 2007). Therefore, Chloroflexi is selected under the influence of the deteriorated environment and can be a disease-inducible microorganism. The abundance of Acidobacteria was evidently higher in the non-diseased group in Dali. Other studies also revealed that it was enriched in the non-continuous cropping (NCC) network (Niu et al., 2016; Chen et al., 2018). It was verified that the abundance of Acidobacteria was negatively correlated with the tobacco disease rate. Acidobacteria are listed as potential probiotic bacteria (Niu et al., 2016) and have genes that encode polyketide synthase and non-ribosomal peptide synthase enzymes, which play a typical role in the synthesis of antifungals and antibiotics (Ward et al., 2009). Acidobacteria, Firmicutes, and Proteobacteria might cooperate in providing other microbial species with carbon sources through the degradation function in the microbial network (Chen et al., 2018). There was an apparent change in the microbial β-diversity between the two groups from Dali and Yuxi. Bray–Curtis PCoA combined with PERMANOVA and Bray–Curtis nMDS combined with ANOSIM analysis showed that the microbial composition in the non-diseased groups was distinctly evident from the diseased groups. Bai et al. (2019) found that the bacterial community structure was strongly influenced by tobacco CC based on hierarchical clustering and PCoAs. Correspondingly, the fungal community was strongly affected after 30 years of tea monoculture based on the unweighted pair group method with arithmetic mean (UPGMA) analysis and PCoA (Arafat et al., 2019). Meanwhile, Arafat et al. (2019) found that low tea production could be ascribed to the decrease in beneficial fungal species (*Mortierella alpine* and *Mortierella elongatula*) and the promotion of pathogenic fungal species (*Fusarium oxysporum* and *Fusarium solani*). This is a common phenomenon occurring in other CC crops, such as potato (Lu et al., 2013), soybean (Bai et al., 2015), and coffee (Zhao et al., 2018). Therefore, the change in the microbial community is a key factor associated with tobacco disease.

The first four dominant KEGG pathways were metabolic pathways, biosynthesis of secondary metabolites, microbial metabolism in diverse environments, and biosynthesis of antibiotics. Bray–Curtis PCoA combined with PERMANOVA and Bray–Curtis nMDS combined with ANOSIM analyses at the KO level presented that the functional genes in the non-diseased groups were separated from the diseased groups. The change in the β-diversity of functional genes was a relevant factor causing tobacco disease in our study. The functional genes concerning MAPK signaling pathway in yeast accumulated in the non-diseased group, and the genes related to ethylbenzene degradation accumulated in the diseased group in Dali (*p* < 10^–4^). MAPK signaling pathway in yeast is listed in the class of environmental information processing, related to the adjustment in nutrient-limiting environment and repair of injuries in the cell wall (KEGG pathway database; Chen and Thorner, 2007). This indicates that the ability of the microorganisms to self-repair and self-adjust has been stimulated and strengthened in the non-diseased group from Dali. Ethylbenzene degradation is in the class of xenobiotics biodegradation and metabolism (KEGG pathway database; Rabus et al., 2002), which means that exotic toxic substances threaten the microbiome in the diseased group from Dali. Simultaneously, the abundance of genes associated with biofilm formation in *V. cholerae* increased significantly in the non-diseased group from Yuxi. The genes related to clavulanic acid biosynthesis accumulated in the diseased group from Yuxi (*p* < 10^–4^). The pathway of biofilm formation in *V. cholerae* belongs to the class of cellular processes, providing microorganisms with advantages of surface colonization and biofilm formation and development (KEGG pathway database; Teschler et al., 2015). Biofilm formation on the plant rhizoplane plays a vital role in the defense system of the plants through inhibiting pathogens’ colonization directly. Therefore, the stronger function of biofilm formation in the microorganisms is an inevitable factor contributing to the non-diseased group in Yuxi. The pathway of clavulanic acid biosynthesis is in the class of biosynthesis of other secondary metabolites (KEGG pathway database). Clavulanic acid is a strong and broader spectrum of inhibition substance to β-lactamase, and the biosynthesis of β-lactamase is the main mechanism of bacterial drug resistance (McGowan et al., 1998). The strengthening in the biosynthesis of clavulanic acid results in the microorganisms being more sensitive to the environment. The weakening in the microbiome’s resilience is a typical feature in the diseased group from Yuxi.

Based on the correlation analysis between the microbial community, functional genes, and soil environmental factors, there was a similar influence pattern of SA and TPA on the microbial community and functional genes, opposite to soil pH. Bai et al. (2019) found that the content of PAs was intimately associated with soil pH. The contents of vanillin, phloroglucinol, *p*-hydroxybenzoic acid, and ferulic acid were negatively related to soil pH. PAs own acidic properties and can accumulate easier under lower pH values. Soil pH is an inevitable crucial factor in soil physicochemical properties influencing microbial composition and diversity in numerous studies (Chen et al., 2018; Bai et al., 2019). It can change microbial osmotic pressure and surface potential directly and PAs contents, the bioavailability of nutrients, and the habitation conditions indirectly (Chen et al., 2018; Bai et al., 2019). The changing pattern of soil pH and SA content between the two groups from Dali was the opposite of that from Yuxi, which was an important reason for the divergent biomarkers of microbiome and functional genes. The different tobacco varieties and natural conditions also contribute to the difference in the two sampling sites. The causes of tobacco CCO are regional from a soil micro-ecology perspective. This phenomenon in other plants and places need to be further studied. Adjusting the soil to the former balanced condition is the basic and crucial concept in alleviating tobacco CCO. Appropriate applications of bio-organic fertilizers, soil conditioners, functional biochar, and tillage regime are suggested according to the specific circumstances.

## Conclusion

The mechanisms of tobacco disease under CC circumstances can be owed to the imbalance and deterioration of soil micro-ecology, which possesses a remarkable regional feature. There were four aspects of mechanisms in our study: the change in soil physicochemical properties, primarily soil pH, OM, available K, CEC, TC/TN contents, and texture; the variance of PAs contents in rhizosphere soil, especially SA; the deficiency of B, K, Mg, and Mn in soil and/or tobacco; and the transformation in microbial community and functional genes from beneficial to harmful in rhizosphere soil. There was an intimate interaction among these four parts. Soil pH and SA had the most significant influence on the composition and structure of microbial communities and functions in rhizosphere soil.

## Data Availability Statement

The datasets presented in this study can be found in online repositories. The names of the repository/repositories and accession number(s) can be found in the article/Supplementary Material. Metagenome data were deposited in the SRA database under accession number PRJNA758121.

## Author Contributions

DC, MW, and XY conceived the original design of the study. KD helped to collect samples. DC, YZ, MW, and SY executed the experiments. DC and JL performed the statistical analysis of the data. DC wrote the manuscript. MM, JL, and XY contributed to the revision of the manuscript.

## Conflict of Interest

KD was employed by the company Yuxi Tobacco Company, Ltd. The remaining authors declare that the research was conducted in the absence of any commercial or financial relationships that could be construed as a potential conflict of interest.

## Publisher’s Note

All claims expressed in this article are solely those of the authors and do not necessarily represent those of their affiliated organizations, or those of the publisher, the editors and the reviewers. Any product that may be evaluated in this article, or claim that may be made by its manufacturer, is not guaranteed or endorsed by the publisher.

## Footnotes

1
http://exon.gatech.edu/meta_gmhmmp.cgi

2
http://www.bioinformatics.org/cd-hit/

3
http://www.genome.jp/kegg/pathway.html
