# Supplementary material for: Succession Pattern in Soil Micro-Ecology Under Tobacco (Nicotiana tabacum L.) Continuous Cropping Circumstances in Yunnan Province of Southwest China
Source: Front Microbiol. 2022 Feb 3;12:785110. doi: 10.3389/fmicb.2021.785110 (PMC8851204; doi:10.3389/fmicb.2021.785110)
Supplement: Supplementary file 1 [file Data_Sheet_1.docx]

Supplementary Materials

# List of Supplementary Materials

1. Supplementary Tables:

- Table S1: The information of sampling sites
- Table S2: The results of sample sequences
- Table S3: The results of sample grouping
- Table S4: The results of gene prediction
- Table S5: The results of non-redundant geneset

2）Supplementary Figures:

- Figure S1: The relative abundance of microbial composition at the species level
- Figure S2: The LDA score (> 2) of the biomarkers among the four groups
- Figure S3: The correlation heatmap between microorganisms at the phylum level **(A)**, or KEGG pathways **(B)** and soil environmental factors

# Supplementary Tables

**Supplementary Table 1.** The information of sampling sites.

| **Sampling sites** | **Locations** | **GPS** | **Altitude (m)** | **Soil type** | **Tobacco variety** | | **Continuous cropping years** |
| --- | --- | --- | --- | --- | --- | --- | --- |
| D1 | Xizhuang village, Xinjie town, Midu county, Dali city | 100°24′37″E, 25°23′3″N | 1712 | Paddy soil | | Honghua  dajinyuan | 3 years |
| D2 | Donghe village, Xinjie town, Midu county, Dali city | 100°25′50″E, 25°23′36″N | 1650 | Paddy soil | | Honghua  dajinyuan | >3 years |
| D3 | Donghe village, Xinjie town, Midu county, Dali city | 100°25′50″E, 25°23′36″N | 1650 | Paddy soil | | Honghua  dajinyuan | >3 years |
| Y1 | Chengbo community, Fenglu town, Chengjiang city, Yuxi city | 102°55′46″E, 24°40′17″N | 1785 | Red soil | | K326 | About 24 years |
| Y2 | Huishijia village, Longjie town, Chengjiang city, Yuxi city | 102°53′35″E, 24°40′33″N | 1771 | Red soil | | K326 | About 24 years |
| Y3 | Longjie village, Longjie town, Chengjiang city, Yuxi city | 102°52′49″E, 24°38′59″N | 1744 | Red soil | | K326 | About 32 years |

D1, D2, and D3 represent the sampling sites showing no, slight, and severe tobacco disease in Dali, respectively. Y1, Y2, and Y3 represent the corresponding sites in Yuxi.

**Supplementary Table 2.** The results of sample sequences.

| **Sample** | **Raw data base (bp)** | **Clean data base (bp)** | **No host clean data base (bp)** | **Number of reads** | **GC (%)** | **Q20 (%)** | **Q30 (%)** |
| --- | --- | --- | --- | --- | --- | --- | --- |
| DR11 | 6,259,635,880 | 5,056,309,762 | 4,619,896,642 | 15,430,074 | 60.56 | 97.4 | 92.83 |
| DR12 | 6,426,348,794 | 5,157,979,116 | 4,995,626,348 | 16,684,202 | 60.65 | 97.72 | 93.55 |
| DR13 | 6,786,536,774 | 5,446,305,790 | 5,341,590,791 | 17,837,840 | 60.75 | 97.46 | 92.97 |
| DR31 | 6,484,419,294 | 5,305,213,330 | 5,246,351,132 | 17,516,233 | 62.96 | 97.27 | 92.54 |
| DR32 | 6,563,801,818 | 5,202,031,659 | 5,177,862,278 | 17,287,491 | 64.44 | 97.62 | 93.3 |
| DR33 | 6,450,936,356 | 5,174,339,078 | 5,127,427,143 | 17,127,791 | 61.48 | 97.72 | 93.58 |
| YR11 | 7,184,883,930 | 5,796,226,033 | 5,752,745,582 | 19,226,887 | 63.87 | 98.57 | 95.62 |
| YR12 | 7,188,501,446 | 5,826,382,037 | 5,790,822,880 | 19,349,275 | 62.13 | 98.19 | 94.66 |
| YR13 | 7,171,348,164 | 5,546,642,939 | 5,458,996,849 | 18,282,736 | 62.86 | 98.48 | 95.44 |
| YR31 | 7,184,191,238 | 5,476,568,650 | 5,277,872,747 | 17,644,192 | 62.37 | 98.37 | 95.15 |
| YR32 | 7,187,822,552 | 5,597,603,740 | 5,494,616,485 | 18,356,521 | 62.32 | 98.28 | 94.89 |
| YR33 | 7,185,451,466 | 5,495,738,677 | 5,383,056,024 | 17,991,510 | 63.2 | 98.54 | 95.53 |

DR1_ and DR3_ (_ = 1, 2, 3) represent the rhizosphere soil in the no disease and severely diseased group from Dali, respectively. YR1_ and YR3_ represent the corresponding samples from Yuxi.

**Supplementary Table 3.** The results of sample grouping.

| **Sample** | **Contig number** | **Total length (bp)** | **Largest length (bp)** | | **N50 (bp)** | **GC (%)** | **Mapped (%)** |
| --- | --- | --- | --- | --- | --- | --- | --- |
| DR11 | 154,319 | 114,573,873 | 50,018 | 695 | | 61.92 | 44.13 |
| DR12 | 149,811 | 109,877,698 | 61,793 | 685 | | 61.77 | 43.71 |
| DR13 | 117,773 | 83,906,414 | 104,644 | 649 | | 61.29 | 37.74 |
| DR31 | 100,277 | 67,951,436 | 59,100 | 636 | | 63.57 | 37.28 |
| DR32 | 128,663 | 89,359,357 | 58,318 | 655 | | 65.36 | 43.82 |
| DR33 | 107,636 | 74,149,712 | 45,162 | 647 | | 61.74 | 43.39 |
| YR11 | 168,755 | 121,077,711 | 25,885 | 669 | | 61.85 | 45.72 |
| YR12 | 183,661 | 148,990,110 | 84,451 | 760 | | 57.71 | 40.29 |
| YR13 | 136,487 | 101,624,777 | 101,968 | 685 | | 61.76 | 48.97 |
| YR31 | 84,962 | 57,598,171 | 37,933 | 635 | | 62.4 | 37.11 |
| YR32 | 193,544 | 164,792,003 | 106,434 | 777 | | 63.28 | 46.21 |
| YR33 | 121,724 | 85,231,226 | 127,716 | 655 | | 63.61 | 40.44 |

DR1_ and DR3_ (_ = 1, 2, 3) represent the rhizosphere soil in the no disease and severely diseased group from Dali, respectively. YR1_ and YR3_ represent the corresponding samples from Yuxi.

**Supplementary Table 4.** The results of gene prediction.

| **Sample** | **Gene number** | **Total length (bp)** | **Average (bp)** | **Max length (bp)** | **Min length (bp)** |
| --- | --- | --- | --- | --- | --- |
| DR11 | 626,303 | 226,436,856 | 361.0 | 6,249 | 102 |
| DR12 | 637,007 | 227,919,273 | 357.0 | 6,666 | 102 |
| DR13 | 588,855 | 205,309,671 | 348.0 | 12,828 | 102 |
| DR31 | 523,480 | 179,005,665 | 341.0 | 4,638 | 102 |
| DR32 | 583,923 | 204,918,426 | 350.0 | 5,256 | 102 |
| DR33 | 557,118 | 190,216,308 | 341.0 | 4,389 | 102 |
| YR11 | 699,717 | 252,345,276 | 360.0 | 7,242 | 102 |
| YR12 | 643,142 | 247,623,528 | 385.0 | 17,316 | 102 |
| YR13 | 618,210 | 220,363,794 | 356.0 | 8,349 | 102 |
| YR31 | 445,679 | 151,520,511 | 339.0 | 3,897 | 102 |
| YR32 | 717,947 | 274,241,352 | 381.0 | 11,475 | 102 |
| YR33 | 548,528 | 192,844,722 | 351.0 | 9,855 | 102 |

DR1_ and DR3_ (_ = 1, 2, 3) represent the rhizosphere soil in the no disease and severely diseased group from Dali, respectively. YR1_ and YR3_ represent the corresponding samples from Yuxi.

**Supplementary Table 5.** The results of non-redundant geneset.

| **Type** | **Geneset number** | **Total length (bp)** | **Average (bp)** | **Max length (bp)** | **Min length (bp)** |
| --- | --- | --- | --- | --- | --- |
| geneset | 6,596,898 | 2,377,237,017 | 360.0 | 17,316 | 102 |

# Supplementary Figures

**
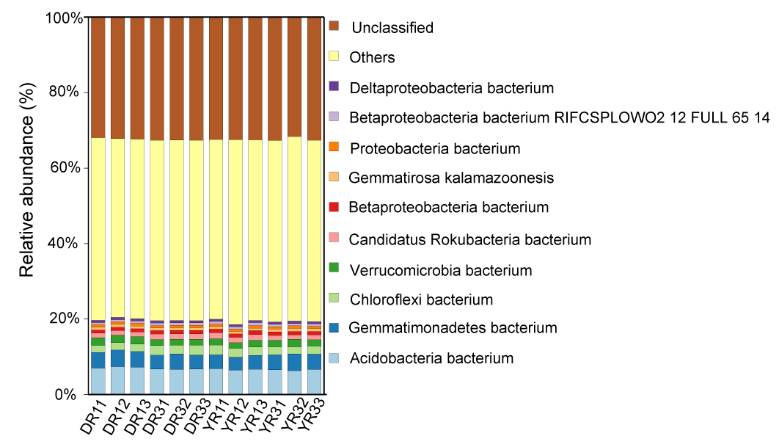
**

**Supplementary Figure 1.** The relative abundance of microbial composition at the species level. Only the first 10 most dominant microorganisms were presented. The remaining was named as “others”. DR1_ and DR3_ (_ = 1, 2, 3) represent the rhizosphere soil in the no disease and severely diseased group from Dali, respectively. YR1_ and YR3_ represent the corresponding samples from Yuxi.

**
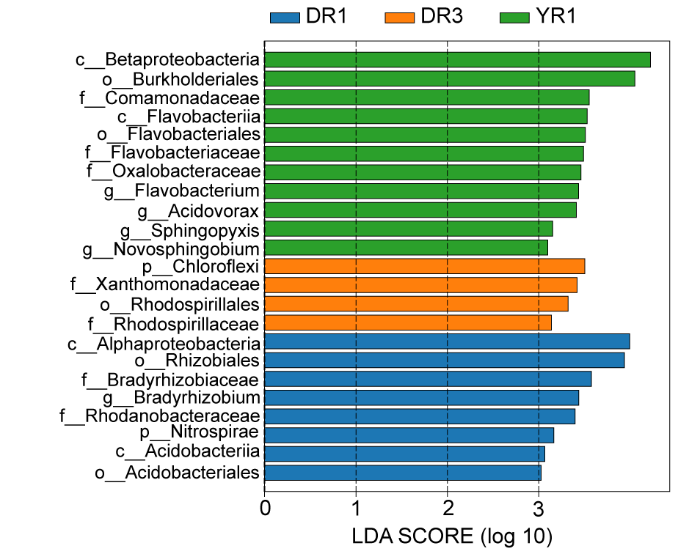
Supplementary Figure 2.** The LDA score (> 2) of the biomarkers among the four groups. The blue, orange, and green taxonomy represent the biomarkers playing an important role in the no disease group from Dali, severely diseased group from Dali, and no disease group from Yuxi, respectively. No such taxonomy exists in the severely diseased group from Yuxi.

**
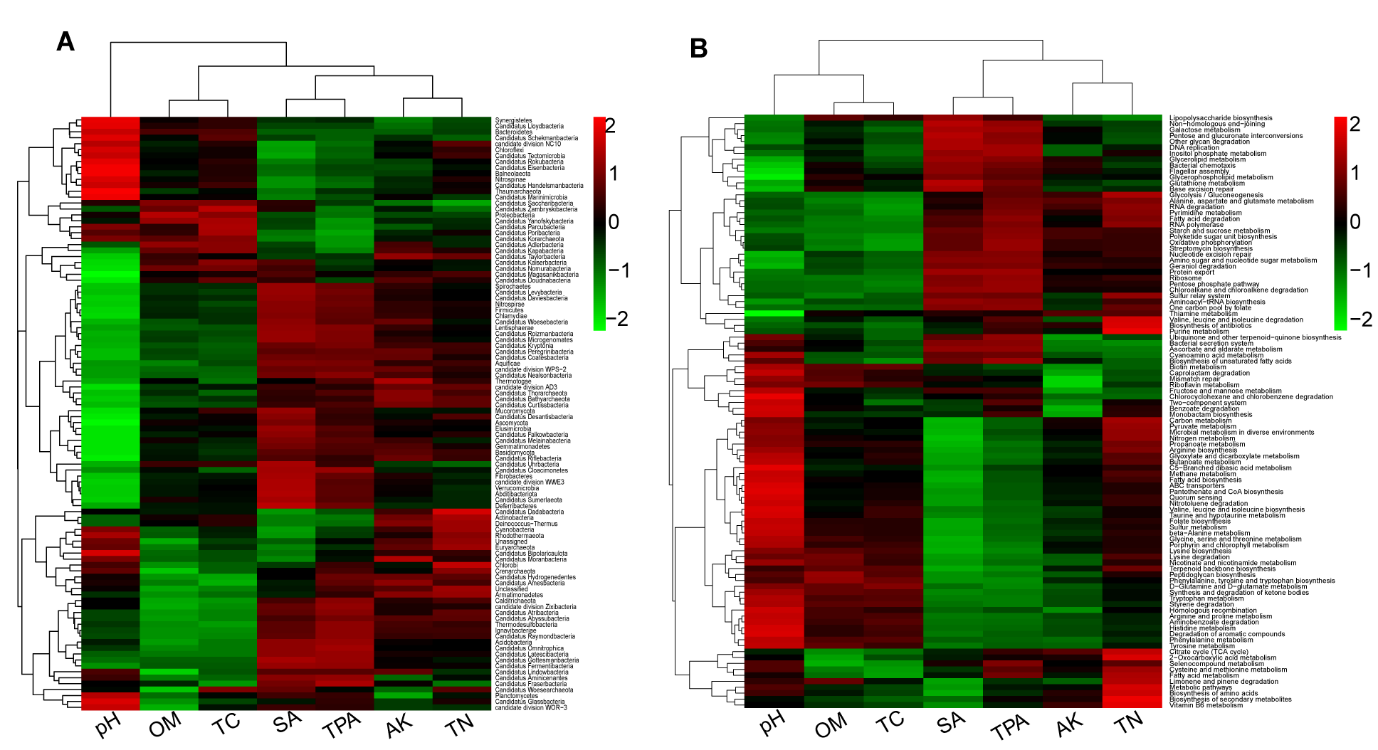
Supplementary Figure 3.** The correlation heatmap between microorganisms at the phylum level **(A)**, or KEGG pathways **(B)** and soil environmental factors. SA, TPA, OM, TC, AK, TN represent the contents of syringic acid, total phenolic acids, organic matter, total C, available K, and total N in rhizosphere soil.
